# Evaluation of genetic parameters affecting the reliability and effectiveness of kinship reconstruction in Forest and Tundra Nenets populations using X-STR markers

**DOI:** 10.18699/vjgb-26-76

**Published:** 2026-09

**Authors:** K.V. Vagaytseva, N.A. Kolesnikov, O.M. Burenkova, I.A. Volkova, O.D. Ruzavina, V.N. Kharkov, V.A. Stepanov

**Affiliations:** Research Institute of Medical Genetics, Tomsk National Research Medical Center of the Russian Academy of Sciences, Tomsk, Russia; Research Institute of Medical Genetics, Tomsk National Research Medical Center of the Russian Academy of Sciences, Tomsk, Russia; Research Institute of Medical Genetics, Tomsk National Research Medical Center of the Russian Academy of Sciences, Tomsk, Russia; Research Institute of Medical Genetics, Tomsk National Research Medical Center of the Russian Academy of Sciences, Tomsk, Russia; Research Institute of Medical Genetics, Tomsk National Research Medical Center of the Russian Academy of Sciences, Tomsk, Russia; Research Institute of Medical Genetics, Tomsk National Research Medical Center of the Russian Academy of Sciences, Tomsk, Russia; Research Institute of Medical Genetics, Tomsk National Research Medical Center of the Russian Academy of Sciences, Tomsk, Russia

**Keywords:** Nenets, X-STR, forensic genetic, kinship testing, linkage disequilibrium, ненцы, X-STR, ДНК-идентификация, реконструкция родственных связей, структура неравновесия по сцеплению

## Abstract

X-chromosomal STR markers (X-STR) are informative tools for kinship reconstruction, particularly in cases where standard autosomal panels have limited discriminatory power: when reconstructing kinship between full sisters, paternal half-sisters, paternal grandmother and granddaughter, maternal aunt and niece. However, the use of X-STR markers in forensic genetics requires consideration of linkage disequilibrium structure and the use of haplotype frequencies in calculating the likelihood ratio (LR). In this work, the genetic structure of Forest and Tundra Nenets populations was assessed using 16 X-STR markers in order to obtain reference values. A sample of 504 Nenets males was analyzed, divided into subethnic groups (Forest and Tundra Nenets), phratries of Tundra Nenets (Haryuchi and Vanuito), as well as geographical local groups (individuals living in the Antipayutinskaya, Gydan and Nakhodka tundras). No statistically significant differences were found between the samples, and the pairwise Fst values (exact test) did not exceed 0.01. The Nenets population demonstrated an intermediate level of genetic diversity typical of Indigenous Siberian populations. The loci DXS8377 (He = 0.910) and DXS10079 (He = 0.839) were found to be the most polymorphic in the Nenets population. Of the 16 markers, 13 were highly informative (PIC > 0.5). The combined power of exclusion (CPE) was 0.99996, which indicates that the panel is highly informative for reconstructing kinship relationships. Pairwise analysis of linkage disequilibrium (LD) revealed statistically significant deviations for 46 pairs of markers out of 120; however, the r2 coefficient for all pairs of alleles did not exceed 0.09, which indicates a weak correlation between alleles. This study provides the reference data of allele and genotype frequencies recommended for use in assessing the likelihood ratio in the reconstruction of kinship relationships. The obtained reference data will contribute to improving the reliability and efficiency of forensic genetic analyses in the Russian Federation.

## Abstract (ru)

X-хромосомные STR-маркеры (X-STR) являются информативным инструментом для реконструкции родственных связей, особенно в случаях, когда стандартные аутосомные панели недостаточно эффективны: при реконструкции родственных отношений между полнородными сестрами, сводными по отцу сестрами, бабушкой по отцовской линии и внучкой, тетей по материнской линии и племянницей. Однако использование X-STR маркеров в ДНК-идентификации требует учета структуры неравновесия по сцеплению и использования частот гаплотипов при расчете отношения правдоподобия (likelihood ratio, LR). В настоящей работе проведена оценка генетической структуры популяций лесных и тундровых ненцев по 16 X-STR маркерам, необходимая для получения референсных значений. Исследована выборка из 504 мужчин ненцев, подразделенная на субэтнические группы (лесные и тундровые ненцы), фратрии тундровых ненцев (Харючи и Вануйто), а также географические локальные группы (индивиды, проживающие в Антипаютинской, Гыданской и Находкинской тундрах). Статистически значимые различия между выборками не обнаружены, попарные значения Fst (exact test) не превысили 0.01. Показан средний уровень генетического разнообразия, характерный для популяций коренных народов Сибири. Наиболее полиморфными в популяции ненцев оказались локусы DXS8377 (He = 0.910) и DXS10079 (He = 0.839). Тринадцать из 16 маркеров были высокоинформативными (PIC > 0.5). Суммарная исключающая способность (CPE) составила 0.99996, что указывает на высокую информативность набора при реконструкции родственных отношений. При попарном анализе структуры неравновесия по сцеплению (LD) статистически значимые отклонения выявлены для 46 из 120 пар маркеров, однако коэффициент r2 для всех пар аллелей не превысил 0.09, что свидетельствует о слабой силе корреляции между аллелями. В статье приведены референсные значения частот аллелей и генотипов, рекомендуемые для использования в оценке отношения правдоподобия при реконструкции родственных связей. Полученные параметры позволят повысить надежность и эффективность генетической экспертизы на территории Российской Федерации

## Introduction

Kinship reconstruction based on the analysis of genetic markers is a key task in forensic genetics. A standard set of autosomal STR markers is a highly effective tool for reconstructing first-degree relationships when biological samples from at least three individuals who are presumably related are available for analysis (for example, a mother, a child, and a presumed father, the so-called “trio”). At the same time, the reliability of this approach is lower in more complex cases due to overlapping ranges of likelihood ratio (LR) values for true relatives and unrelated individuals (Ge, Budowle, 2020).

To increase the informativeness in such cases, it is common to use additional genetic markers: STR, SNP, and InDEL. Markers localized on the X chromosome are particularly informative for some variants of pedigree reconstruction. Due to the specific inheritance pattern of the X chromosome and hemizygosity in males, X-chromosomal markers can be used to establish a relationship between an alleged father and daughter even when the biological sample of the man himself is unavailable; in such cases, a sample from the man’s mother may be included in the forensic genetic examination. It is shown that the inclusion of 12 X-STR markers in the analysis leads to an increase in the effectiveness of forensic genetic examination for the following types of kinship pairs: full sisters, paternal half-sisters, paternal grandmother–granddaughter, as well as maternal aunt–niece, maternal aunt–nephew. For other kinship categories, the effect of including X-STR is comparable to the inclusion of additional autosomal STR markers, and in some cases (for example, maternal uncle–nephew) it is inferior to it (Zhang et al., 2021a). At the same time, the use of X-chromosome markers in the practice of forensic genetics leads to a complication of statistical calculations due to the linkage between loci.

According to the recommendations of the International Society for Forensic Genetics (ISFG), when marker sets containing linked loci are used, haplotype frequencies should be applied in probabilistic assessments, and the structure of linkage disequilibrium should be taken into account (Tillmar et al., 2017). However, neither of these parameters is universal: they vary depending on the genetic and demographic history of the population. Therefore, before introducing any set of X- STR markers into forensic practice for a specific population, it is necessary to conduct population genetic studies.

The aim of the present study is to analyze the genetic structure of the Forest and Tundra Nenets populations using 16 X- STR markers to form a reference base necessary to increase the effectiveness of kinship reconstruction in the practice of forensic genetics.

This study was approved by the Biomedical Ethics Committee of the Research Institute of Medical Genetics, Tomsk National Research Medical Center of the Russian Academy of Sciences (Protocol No. 19 of October 13, 2023).

## Materials and methods

The study was based on a sample of Nenets males (760 individuals) from the biobank “Biobank of the Population of Northern Eurasia” of the Research Institute of Medical Genetics. Individuals identified as close relatives (father and son, brothers, uncle and nephew) were excluded from this sample. Kinship, population affiliation, place of birth, and place of residence were established based on a questionnaire. After filtering, the sample size was reduced to 504 individuals. The final sample comprises several subsamples: Forest Nenets (46 samples) and Tundra Nenets (458 samples); Tundra Nenets of the Haryuchi phratry (288 samples) and Tundra Nenets of the Vanuito phratry (158 samples); Nenets living in the Antipayutinskaya (107 samples), Gydan (56 samples), and Nakhodka (59 samples) tundras. All samples originate from the Yamalo-Nenets Autonomous Okrug. Venous blood samples were collected after obtaining written informed consent from the donors.

Genotyping of samples using X-STR markers was performed by multiplex polymerase chain reaction followed by analysis of the products using a Nanophore 05 genetic analyzer (Syntol, Russia). The primer sequences and marker information are presented in Y. Zhang et al. (2021b). The PCR reaction was carried out using a reaction mixture and an activator from Gordiz (Russia). The PCR mixture per sample included 2 μL of activator, 2 μL of reaction mixture, 1 μL of DNA sample with a concentration of more than 10 ng/μL, and 5 μL of deionized water. The polymerase chain reaction was performed under the following conditions: 95 °C for 5 min; 36 cycles of 95 °C for 30 s, 60 °C for 45 s, and 72 °C for 45 s; final elongation: 72 °C for 4 min. Fluorescent peaks were evaluated using the GeneMarker software. Allele assignment was performed by comparing fragment sizes with those of the 9947DNA control sample (Applied Biosystems, USA) and the MK01 control sample from Gordiz (Russia). The experimental studies were conducted at the Center for the Collective Use of Scientific Research Equipment “Medical Genomics” (Research Institute of Medical Genetics, Tomsk National Research Medical Center).

Polymorphism information content (PIC), expected heterozygosity (He), and power of exclusion (PE) were calculated using the ChrX-STR.org online calculator (ChrX-STR.org database, accessed January 10, 2026). The combined power of exclusion (CPE) for the entire set of markers (SoftGenetics LLC, 2012), the effective number of alleles (Ne), and allele frequencies were calculated in a standard manner using the MS Excel software package. Analysis of molecular variance (AMOVA), evaluation of pairwise Fst and Rst values, assessment of linkage disequilibrium (LD) structure, and estimation of haplotype frequencies were performed using the Arlequin v.3.5 software tool (Excoffier, Lischer, 2010; Gusmão et al., 2025). Principal component analysis (PCA) was performed in the R environment using the FactoMineR (PCA function) and factoextra visualization packages (Lê et al., 2008).

## Results

**Genetic differentiation of populations**

The first stage of the study was to evaluate the possibility of pooling the Nenets samples into a metapopulation. To this end, a principal component analysis (PCA) plot was constructed to visualize the general genetic relationships between individuals (see the Figure).

**Fig. 1.**
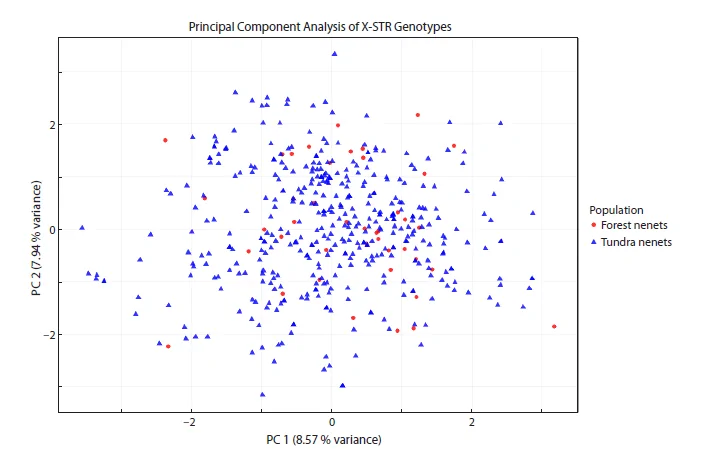
Distribution of forest and tundra samples in the space of the first two main components

The distribution of individuals in the space of the first two principal components (which together account for 16.51 % of the total variability) does not demonstrate the formation of isolated clusters corresponding to the sample of Forest or Tundra Nenets. We used three indicators to evaluate genetic differentiation using F-statistics. Two of them characterize the strength of differentiation (Fst, σμ2), and one (exact test) characterizes the statistical significance of differentiation (Table 1).

**Table 1.**
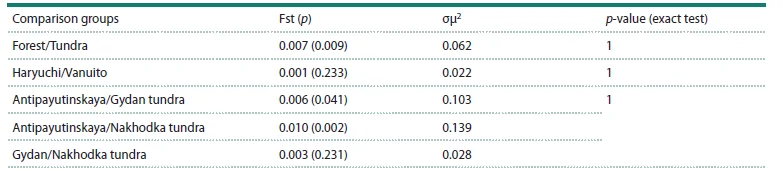
Indicators of genetic differentiation of Nenets populations Notе. Fst (p) are the pairwise values of Fst (population comparisons), the significance level values are shown in parentheses; σμ2 is the genetic distance between microsatellite loci; p-value is the significance level value for an accurate population differentiation test.

The pairwise Fst values in all comparisons are very low (from 0.001 to 0.010), which indicates weak genetic subdivision of the populations, explained by intensive gene flow between populations and a relatively recent divergence time. The exact test showed no statistically significant differences between the populations.

**Genetic diversity and identification potential**

Since pairwise Fst values among the studied samples did not exceed 0.01, the level of genetic differentiation was considered negligible, following common practice in population genetic studies (Gusmão et al., 2025), it was decided to treat the samples as a single metapopulation. All samples were combined into a single metapopulation (504 samples) to assess the genetic diversity and identification potential of X-STR markers (Table 2).

**Table 2.**
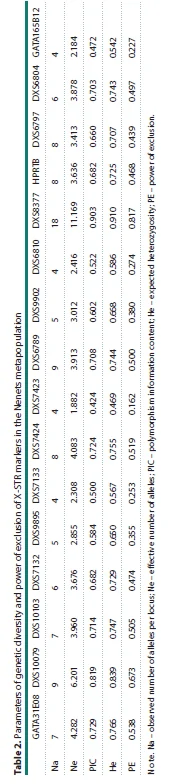
Parameters of genetic diversity and power of exclusion of X-STR markers in the Nenets metapopulation Notе. Na – observed number of alleles per locus; Ne – effective number of alleles; PIC – polymorphism information content; He – expected heterozygosity; PE – power of exclusion.

The calculation results showed that the Nenets population demonstrates average genetic diversity, which is typical for the Indigenous peoples of Siberia who are in relative isolation (Vagaitseva et al., 2015, 2025). As can be seen from Table 2, the effective number of alleles varies from 1.882 (DXS7423) to 11.169 (DXS8377), and the expected heterozygosity (He), from 0.469 (DXS7423) to 0.910 (DXS8377), which indicates a pronounced variability in the level of polymorphism between loci. According to the classification of D. Botstein et al. (1980), 13 out of the 16 markers (81.3 %) are highly informative (PIC > 0.5), including the most polymorphic DXS10079 and DXS8377 (PIC > 0.8). The combined power of exclusion (CPE) of the entire set of markers was 0.99996. The values obtained are comparable to those typical for other Indigenous peoples of Siberia, but they are inferior to those in European populations (Vagaitseva et al., 2015; Mršić et al., 2017).

**The linkage disequilibrium**

Localization of the analyzed markers on the X chromosome both gives them advantages for the reconstruction of kinship and complicates the analysis. The alleles of the linked loci are inherited together, forming haplotypes. Ignoring the linkage disequilibrium structure in probabilistic calculations, in which the observed frequency of a haplotype in a population significantly deviates from the product of the frequencies of its constituent alleles, can lead to systematic errors.

Differences in the genetic and demographic history of a population form a unique pattern of linkage disequilibrium, which requires an empirical assessment for each specific population and does not allow extrapolating the data obtained from the analysis of some samples to other populations. To empirically assess the linkage disequilibrium structure between the considered X-STR loci in the Nenets metapopulation, a pairwise analysis was performed with the calculation of two generally accepted indicators: the significance level for each pair of loci ( p-value) and the correlation square r2. The matrix of p-values (Table 3) shows many statistically significant associations. With a threshold significance level of p < 0.05, 46 pairs out of 120 loci showed statistically significant LD. At the same time, the r2 values for all pairs of alleles did not exceed 0.09. This means that despite the statistical significance, the correlation strength is low.

**Table 3.**
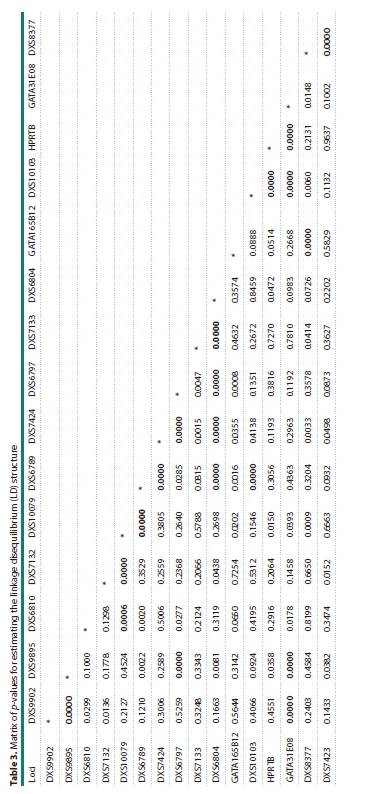
Matrix of p-values for estimating the linkage disequilibrium (LD) structure

**Reference data**

The reference data necessary for probabilistic calculations in the reconstruction of kinship in the Nenets population are presented in the Supplementary Materials1. The Supplementary Tables show the frequencies of alleles of 16 X-STR loci and the frequencies of haplotypes of loci located in the same linkage group: II linkage group (DXS7132–DXS10079), III linkage group (DXS10103–HPRTB), IV linkage group (DXS8377–DXS7423) (Database ChrX-STR.org, accessed January 10, 2026).

Supplementary Materials are available in the online version of the paper: https://vavilov.elpub.ru/jour/manager/files/Suppl_Vag_Engl_30_5.xlsx


## Discussion

The Nenets are an Indigenous people of the Russian North, belonging to the Uralic language family. They are divided into two main sub-ethnic groups, the Forest and Tundra Nenets, which differ in their geographical distribution, cultural characteristics, and dialects. The Tundra Nenets include two main phratries: Haryuchi (Haryutsa) and Vanuito (Vanueta) (Khomic, 1996). The analysis of the samples under consideration by Y-chromosomal markers conducted by V.N. Kharkov et al. demonstrated a high degree of genetic differentiation, fully corresponding to the clan and phratry organization (Zvénigorosky et al., 2020; Kharkov et al., 2025). The X-STR analysis results obtained in our study, in contrast, demonstrated the absence of significant differences between the samples (exact test, p = 1). Such differences in the level of genetic differentiation indicate an asymmetric gene flow due to social organization.

The key characteristics of the Nenets marriage system are patrilineality, exogamy, and patrilocality (Khomic, 1996). Unlike men, women actively moved between clans, phratries, and even sub-ethnic groups. Our results are consistent with the demographic history of the Nenets population. The results of the X-STR marker study demonstrated an intermediate level of genetic diversity in the analyzed Nenets population. This is due to the founder effect during human settlement of Northern Eurasia, and the subsequent genetic drift in isolated groups.

Pairwise Fst analysis showed a low level of genetic differentiation, values not exceeding 1 %. Of the five pairwise comparisons, statistical significance ( p < 0.05) was observed in only two. At the same time, the differences between the samples of the Antipayutinskaya and Nakhodka tundras (Fst = 0.010, p = 0.002) exceed those between the Forest and Tundra Nenets (Fst = 0.007, p = 0.009). For comparison, in our other study using 18 X-STR markers, weak statistically significant differences (Rst = 0.007, p = 0.002) were also revealed between two other populations of Siberia (Telengits and Altai-Kizhi), and the level of genetic diversity (He) in Altai populations was higher (mean He in the Nenets population = 0.697, in the Altai-Kizhi population = 0.726, and in the Telengits population = 0.723) (Vagaytseva et al., 2025). The obtained value of the combined power of exclusion (CPE = 0.99996) confirms that the presented panel of 16 X-STR markers is quite informative and can be used for pedigree reconstruction in the Nenets population. It is shown that when reconstructing the kinship of individuals from the Nenets population, it is possible not to take into account the territory of residence of the analyzed individuals, due to the lack of statistically significant differentiation between the analyzed Nenets groups (exact test p = 1; Fst < 1 %). In this paper, the analyzed sample of Forest Nenets is small (46 samples), which may affect the robustness of the results obtained. Nevertheless, the presented data are the most comprehensive characterization of the genetic structure of the Nenets population by X-STR markers to date.

## Conclusion

The present study provides reference data on 16 X-STR markers for the Nenets population, which are necessary to increase the efficiency and reliability of forensic genetic examination. This is especially important for populations of Indigenous peoples of Siberia, since the low level of genetic diversity of neutral markers used in forensic genetics makes standard autosomal STRs (aSTRs) less informative (Zvénigorosky et al., 2020). The obtained parameters can be used in the practice of forensic genetics to increase the reliability of forensic genetic examination in the Russian Federation

## Conflict of interest

The authors declare no conflict of interest.
